# Cardiac magnetic resonance radiomics: basic principles and clinical perspectives

**DOI:** 10.1093/ehjci/jeaa028

**Published:** 2020-03-06

**Authors:** Zahra Raisi-Estabragh, Cristian Izquierdo, Victor M Campello, Carlos Martin-Isla, Akshay Jaggi, Nicholas C Harvey, Karim Lekadir, Steffen E Petersen

**Affiliations:** j1 Department of advanced cardiovascular imaging, William Harvey Research Institute, NIHR Barts Biomedical Research Centre, Queen Mary University of London, Charterhouse Square, London EC1M 6BQ, UK; j2 Barts Heart Centre, St Bartholomew’s Hospital, Barts Health NHS Trust, West Smithfield, EC1A 7BE London, UK; j3 Departament de Matemàtiques & Informàtica, Universitat de Barcelona, Gran Via de les Corts Catalanes, 585, 08007 Barcelona, Spain; j4 MRC Lifecourse Epidemiology Unit, University of Southampton, Tremona Road, Southampton SO16 6YD, UK; j5 NIHR Southampton Biomedical Research Centre, University of Southampton and University Hospital Southampton NHS Foundation Trust, Tremona Road, Southampton, SO16 6YD, UK

**Keywords:** radiomics, texture analysis, cardiac magnetic resonance, image-based diagnosis, machine learning

## Abstract

Radiomics is a novel image analysis technique, whereby voxel-level information is extracted from digital images and used to derive multiple numerical quantifiers of shape and tissue character. Cardiac magnetic resonance (CMR) is the reference imaging modality for assessment of cardiac structure and function. Conventional analysis of CMR scans is mostly reliant on qualitative image analysis and basic geometric quantifiers. Small proof-of-concept studies have demonstrated the feasibility and superior diagnostic accuracy of CMR radiomics analysis over conventional reporting. CMR radiomics has the potential to transform our approach to defining image phenotypes and, through this, improve diagnostic accuracy, treatment selection, and prognostication. The purpose of this article is to provide an overview of radiomics concepts for clinicians, with particular consideration of application to CMR. We will also review existing literature on CMR radiomics, discuss challenges, and consider directions for future work.

## Introduction

Cardiac magnetic resonance (CMR) is the reference imaging modality for assessment of cardiac structure and function; accordingly, its use in clinical practice is increasingly widespread. Clinical reporting of CMR is mostly reliant on qualitative descriptors and basic geometric quantifiers. Existing quantitative measures of tissue character, such as T1/T2 mapping are limited by ongoing technical challenges and poor discriminatory power due to broad overlap between health and disease. As such, currently, much of the information available from CMR images is not optimally utilized. There are shortcomings with this approach. For instance, through existing analysis approaches, it may not be possible to distinguish with certainty between disease entities that appear morphologically similar, such as hypertensive heart disease and hypertrophic cardiomyopathy (HCM) or athletic cardiac remodelling and dilated cardiomyopathy. Such distinctions are important, as management for these conditions is very different. Furthermore, our ability to accurately predict important outcomes is suboptimal. For instance, many patients with prophylactic intracardiac defibrillators based on low ejection fraction never require therapies from their device,^1^ whilst only 30% of sudden cardiac death patients would qualify for a primary prevention device based on current guidelines.^2^ Therefore, novel imaging biomarkers that improve the diagnostic accuracy and predictive capabilities of CMR are needed and highly desirable.

Radiomics is a novel image analysis technique, whereby digital images are converted to data that can be analysed to derive multiple numerical quantifiers of shape and tissue character—referred to as ‘radiomics features’. It has been shown that disease conditions or clinical outcomes may be identified with high accuracy based on the features observed.^3^ Thus, radiomics features may be used as predictor variables in statistical models for diagnosis or outcome prediction. Radiomics models have had notable success in oncology, where their utility in classification of tumours,^4^ prediction of treatment response,^5^^,^^6^ and prognostication^7^ has been demonstrated in multiple cohorts.

Within cardiology, experience with radiomics is limited. Early descriptions from echocardiography demonstrate the utility of radiomics models in distinguishing conditions such as cardiac amyloid^8^ and haemochromatosis.^9^ However, application of radiomics to echocardiography was halted due to difficulties with reproducibility. More recently, there has been interest in application of radiomics analysis to cardiac computed tomography images, where radiomics analysis for characterization of coronary plaques and perivascular fat has produced promising results.^10^^,^^11^ Limited proof-of-concept studies have demonstrated the feasibility and potential clinical value of CMR radiomics.^12–21^ Radiomics analysis can be applied to existing routinely acquired images and does not require dedicated acquisitions or significant post-processing. As such, it has real potential to transition into the routine clinical workflow as an adjunct to conventional CMR measures. CMR radiomics has the potential to transform our approach to defining image phenotypes, and through this, improve diagnostic accuracy, treatment selection, and prognostication.

The purpose of this article is to provide an overview of radiomics concepts for clinicians, with particular consideration of application to CMR. We will review basic radiomics concepts and workflow (*Figure 7*), existing literature on CMR radiomics, and discuss challenges and directions for future work.

## The radiomics workflow

### Image acquisition

Radiomics analysis can be applied to standard, routinely acquired clinical images. There is no requirement for dedicated acquisitions or imaging protocols. Any image from the CMR scan can be selected for radiomics analysis; however, the short-axis stack is the most convenient as existing endocardial and epicardial contours can be used to define the regions of interest (ROI), avoiding extra segmentation steps. Whilst still images are used for radiomics analysis, information relating to motion may be gauged through analysis of temporally related images, e.g. analysis of images in end-systole and end-diastole, or assessment of images from all phases of the cardiac cycle.

### Volume segmentation

**Figure 1:**
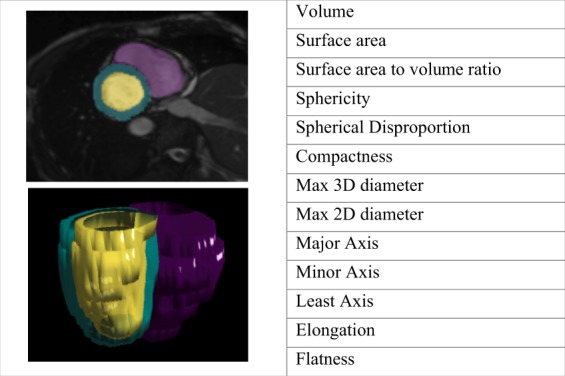
Image mesh derived from segmented volume and selected radiomics shape features.^a^^a^An image mask is derived from contours of the ventricles in the short-axis cine stack. The mask is an approximation to the 3D shape of the contour. In this example, the blood pool of the right (purple) and left (yellow) ventricles, and the left ventricular myocardium (turquoise) are represented. Radiomics shape features are derived from these masks and include conventional and more advanced geometric quantifiers.

Once the image to be used is selected, the area for radiomics analysis is defined by contouring an ROI. The ROI may be a limited area [a single region of suspected abnormality within the left ventricular (LV) myocardium] or multiple areas. Typically, we delineate the endocardial and epicardial borders of the left ventricle and the endocardial border of the right ventricular (RV) in one phase of the short-axis stack—this defines the boundaries of the LV myocardium, and the RV/LV blood pool (three areas, *Figure 1*). Once defined, you may apply radiomics analysis to the any of these regions. As the radiomics features are extracted from the defined areas, variations in contouring that alter the ROI will change the values of the radiomics features. Therefore, it is key to have a consistent contouring technique defined through a standard operating procedure (SOP). Our approach is to use standard epicardial and endocardial contouring of the ventricles as would be used for conventional volume quantification according to a previously described SOP.^22^ We advocate automated contouring with limited manual correction as this produces the most reproducible segmentation. Automated segmentation can also allow rapid contouring of the entire cardiac cycle, which may yield more information in comparison to analysis of a single image/slice or analysis at two time points (e.g. end-systole/diastole). Automated segmentation is now integrated into many commonly used CMR post-processing packages and will likely become increasingly common-place with continued advancement of artificial intelligence technologies.

### Radiomics feature extraction

**Figure 2:**
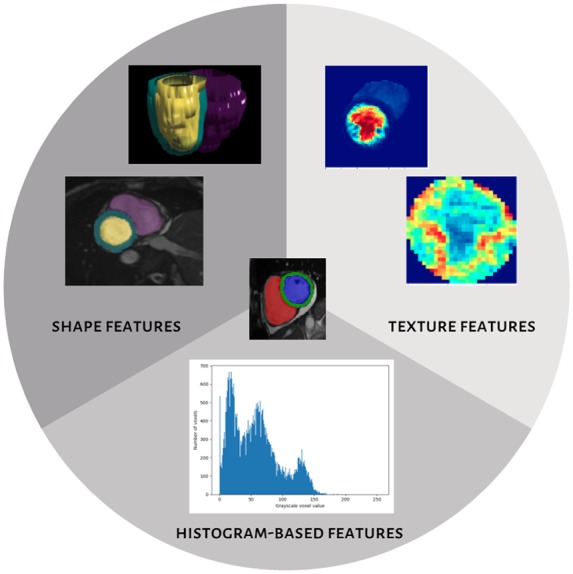
Summary of the types of radiomics features.^a^^a^*Shape features*: An image mask is an approximation of the 3D shape of the region of interest, in this case, it is derived from the ventricular contours. The radiomics shape features are derived from this image mask and include conventional and more advanced geometric quantifiers. *Texture features*: Texture features are derived by assigning a signal intensity level to each voxel in the region of interest and considering the pattern and relationships between different voxel signal intensities through application of various mathematical processes. *Histogram-based features*: The signal intensities observed in the analyzed region of interest may be described by plotting a histogram with voxel signal intensity value on the *x* axis and frequency on the *y* axis. Summary statistics derived from the histogram such as mean, median, and standard deviation may be used to describe the global signal intensity distribution.

Extraction of radiomics features from the segmented ROI can be performed through dedicated pipelines developed by individual centres or using open-source packages, such as Py-radiomics.^23^^,^^24^ Radiomics features include numerical quantifiers of the geometry of the ROI, the global signal intensity (SI) distribution, and the spatial complexity of SIs within the segmented volume (*Figure 2*).

### Radiomics shape features

Radiomics shape features quantify the 3D size and shape of the segmented volume. Shape features are derived from an image mesh/mask approximating the defined edges of the ROI,^25^ in our case, this would be the endocardial contours (*Figure 1*). Radiomics shape features include conventional indices (e.g. volume), as well as additional parameters, such as surface area and dimensions in multiple planes. There are also descriptors of the overall shape of the ROI, such as compactness, sphericity, elongation, and flatness.

### Radiomics signal intensity-based features (texture analysis)

The remainder of the analysis is focused on describing the distribution and pattern of SIs within the segmented ROI, which is a slightly more abstract concept in comparison to the shape analysis. It is thought that the pattern of SIs in the ROI may reflect underlying tissue characteristics which would indicate particular diseases. For instance, a heterogeneous SI pattern in the myocardium may reflect irregular arrangement of myofibrils which may in turn indicate underlying pathology such as HCM. The purpose of radiomics texture analysis is to recognize and quantitatively describe various SI patterns within the selected ROI. This is achieved by numerically defining the SIs within the segmented volume and describing observed patterns using mathematical definitions. These SI-based texture features are often given descriptive names such as ‘busyness’ or ‘randomness’ to denote the underlying property they aim to represent. The ultimate goal with radiomics modelling is to define unique SI patterns (radiomics signatures) for important cardiac diseases, which may be used to improve diagnostic accuracy or perhaps allow automated generation of diagnoses in a manner that would not be possible through qualitative inspection of images.

**Figure 3:**
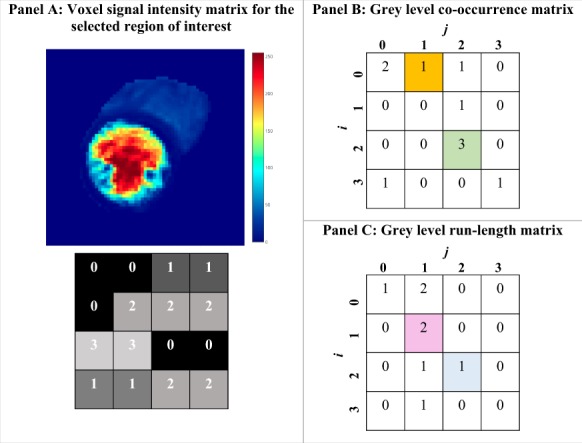
Simplified worked example of grey-level co-occurrence and run–length matrices. (*A*) A signal intensity (SI) level is assigned to each voxel within the selected region of interest and tabulated in a matrix. In this example, we suppose a 4  × 4 matrix with 16 voxels at four signal intensity levels. (*B*) Grey-level co-occurrence matrix corresponding to Panel *A*. In this example, we will consider any voxel with SI *j*, that appears to the right of a reference voxel with SI of *i*. For example to fill the orange cell (*j* = 1, *i* = 0), we count one instance in the Panel *A* matrix, where a voxel with signal intensity level of 1 (*j* = 1) appears to the right of a voxel with signal intensity of 0 (*i* = 0). Hence, we fill the cell in the GLCM matrix with the number one. Similarly, for the green cell (*j* = 2, *i* = 2), we observe that in the whole of the matrix in Panel *A*, there are three instances where a voxel with SI value of 2 (*j* = 2) appears to the right of a voxel with the SI of 2 (*i* = 2), hence cell (2, 2) is filled with the number 3. In the same way, the rest of the matrix is completed. (*C*) Grey-level run-length matrix corresponding to Panel *A*. To complete this matrix, we consider the number of times Panel *A* contains an uninterrupted train of length *j* (measured in number of voxels, e.g. one voxel=run length of zero, two voxels=run length of one) with SI of *i*. For example, consider the pink cell (*j* = 1, *i* = 1); in the matrix of Panel *A*, we count two instances of voxels with SI of 1 (*i* = 1) occurring in an uninterrupted run of length 1 (*j* = 1), hence the cell is filled with the number 2. Similarly, consider the blue cell (*j* = 2, *i* = 2), in our SI matrix, we count one instance where SI of 2 (*i* = 2) appears in an uninterrupted run of three voxels (*j* = 2), hence this cell is filled with the number 1.

The first step to performing radiomics texture analysis is construction of a ‘SI matrix’, whereby each voxel within the ROI is assigned a number (level/value) depending on the intensity of signal in that voxel. The SI value for every voxel in the ROI is then tabulated to form a simple matrix (*Figure 3A*). All SI-based radiomics features are derived from analysis of the SI matrix.

#### Descriptors of global signal intensity

**Figure 4:**
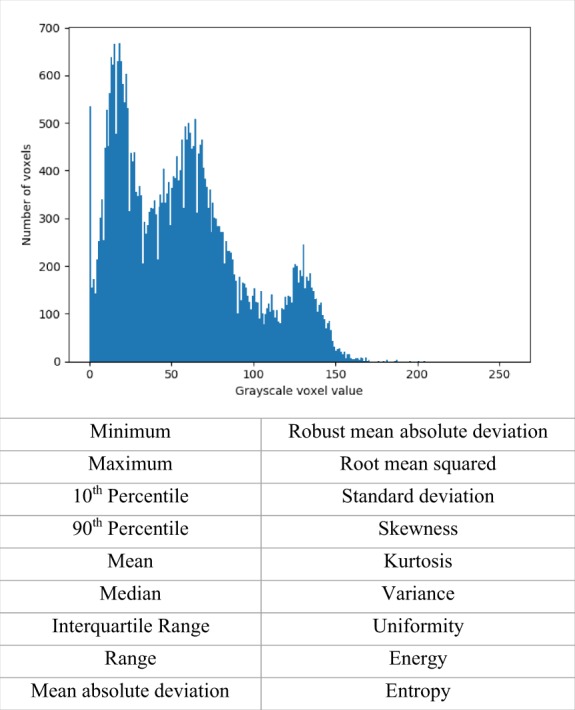
Selected first-order histogram-based statistics to describe global signal intensity distribution within the selected region of interest.^a^^a^The figure depicts a histogram of signal intensity values observed in the region of interest selected for radiomics analysis. The *x* axis represents the signal intensity value of the voxels within the region of interest and the *y* axis the frequency with which these signal intensities values are observed. Below the figure, we present a selection of the summary statistics derived from the histogram (histogram-based statistics) that describe the global signal intensity distribution within the analysed region.

The most straightforward analysis of the SI matrix involves creating a histogram of the SI levels identified in the ROI and the frequency with which they are observed. From this, first-order histogram-based statistics can be computed (*Figure 4*). This includes simple descriptive summary statistics, such as mean, median, and standard deviation, as well as less familiar measures of skewness, kurtosis (pointiness), and entropy (randomness or disorder). These histogram-based texture features provide a global summary of the SIs within the segmented volume; however, they do not describe the relationship of the voxel SIs to each other.

#### Descriptors of spatial distribution of signal intensities

In order to consider the relationship of neighbouring voxel SIs, more complex mathematical approaches to analysis of the SI matrix are required. These features are derived by considering the spatial distribution of SIs within the ROI and aim to quantify heterogeneity, repeatability, and complexity of the SI matrix.^26^^,^^27^ They are computed through application of various mathematical processes to new matrices which are constructed according to specified rules from the original SI matrix. For instance, a common approach for considering the relationship between voxel pairs is through construction of a grey-level co-occurrence matrix (GLCM). The GLCM is constructed by tabulating the frequency of different SI pairings occurring within the SI matrix (*Figure 3B*). Different mathematical processes are then applied to the GLCM to compute, according to agreed definitions, measures, such as angular second moment (homogeneity), contrast (local variation), and entropy (disorder or randomness).^28^ These features reflect the probability of certain SI pairings, the level of grey-level variation interdependencies, and the extent of disorder within the ROI. The grey-level run length matrix (GLRLM) is another commonly constructed matrix.^29–32^ It can be used to consider the spatial relationship of any number of voxels (not just pairs). A GLRLM is constructed by recording the number of times a voxel with a specific SI is seen in an uninterrupted run within the image SI matrix in a specified direction (*Figure 3C*). The GLRLM is used to calculate a number of features such as short-run emphasis, run-length non-uniformity, and run entropy.

A number of other matrices, tabulated according to different rules, are also available for calculation of additional features [grey-level size zone matrix (GLSZM), grey-level difference matrix (GLDM), and neighbouring grey tone difference matrix (NGTDM)]. Supplementary data online, *Table S1* shows a selection of available grey-level matrices and their related features. For each ROI many matrices may be constructed with consideration of matrix rules in different directions within the three-dimensional space of the segmented volume.

### Pre-processing options

In some cases, there is need for pre-processing of images to ensure that the observed variations in brightness and contrast reflect differences in tissue character rather than differences in scaling or matrix size. This involves processes such as grey-level normalization, non-uniformity corrections, and reshaping of images. In addition, filters/transforms may be applied to the original images to derive ‘filtered/transformed’ images that may then be used for radiomics analysis as per the standard workflow.

### Feature selection and dimensionality reduction

The process of feature extraction will yield a large number of radiomics features (100–1000 s). The aim is to use the extracted features as predictor variables within a statistical model for disease classification or outcome prediction. The number of extracted radiomics features often far exceeds the sample size of cohorts used for model building. Using all the extracted features in a statistical model would lead to overfitting, where the model corresponds too closely to the training dataset, such that it picks up noise and performs poorly in internal and external validation. Therefore, we need to select a reduced number of features for model building. This process of ‘feature selection’ occurs after extraction of features from the test dataset (the sample of cases from which the model will be built), but precedes the model building stage (*Figure 7*). The purpose of feature selection is to identify the optimal set of radiomics features to be taken forward for model building. We would aim to include in the model features that are most informative and robust and remove those that are unstable or provide repetitive information.

**Figure 5:**
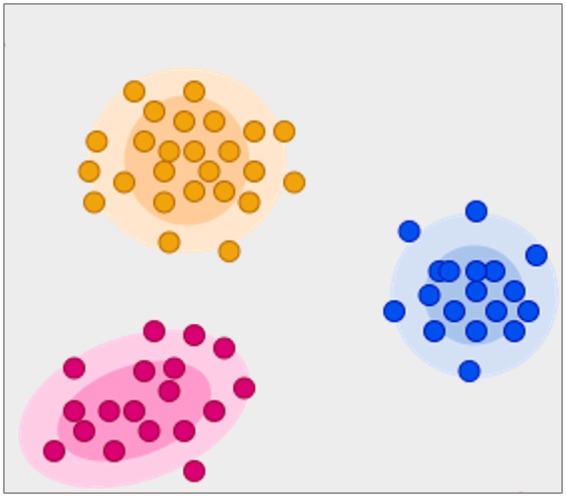
Clustering as a method for feature selection. In this example, the clustering algorithm has grouped radiomics features into three clusters (red, orange, and blue) based on high inter-cluster correlation and low extra-cluster correlation. The algorithm will then select the most representative feature from each cluster and remove the remaining features.

Robustness of features can be assessed through test–retest, with removal of those with poor repeatability. It is expected that many radiomics features will reflect duplicate information; for instance, consider an ROI in the shape of a sphere—the volume, diameter, and surface area will be highly correlated and inclusion of all these shape features is unnecessary. Various methods may be used to identify such highly correlated features and select the most informative.^33^^,^^34^ The most popular approaches are unsupervised machine learning methods such as clustering and principal component analysis. Clustering algorithms group features into clusters based on high correlation with each other (inter-cluster correlation) and low correlation with other features (extra-cluster correlation). The algorithm then identifies the most defining feature from each cluster for inclusion in the model and removes the rest (*Figure 5*). Principal component analysis through different methods reduces the extracted features to a subset that provides nearly as much information as the whole feature-set.^35^

Feature selection requires substantial computational power and time and can be a rate-limiting step in building of radiomics models. Regardless of the method used for feature selection, the common goal is identification of a reduced set of radiomics features that are robust, informative, and non-redundant for inclusion in the predictive model.

### Model building

**Figure 6:**
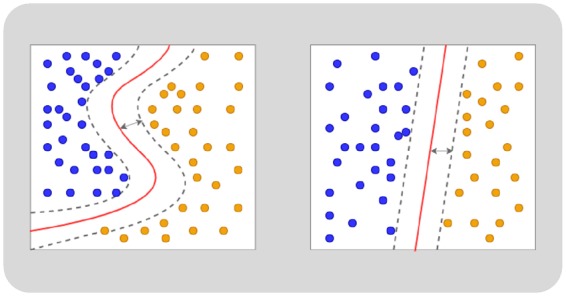
Simplified depiction of model building using a support vector machine. The support vector machine (SVM) algorithm identifies all potential hyperplanes that could separate the two data categories (orange vs. blue). The plane offering the greatest margins with the categories is selected as the optimal model. Both linear (right) and non-linear models (left) may be considered.

**Figure 7:**
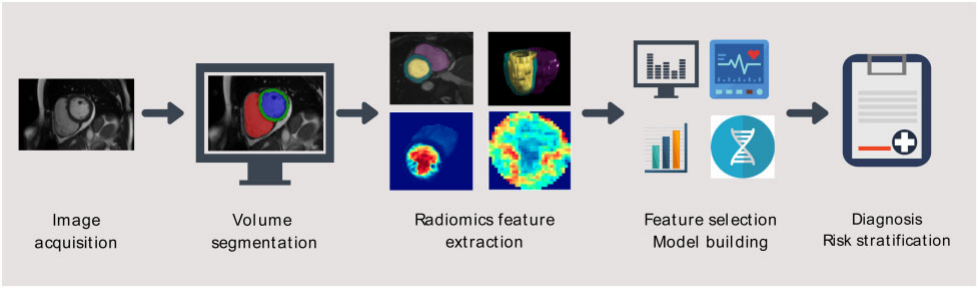
Stepwise depiction of the cardiac magnetic resonance radiomics workflow. *Image acquisition*: Routinely acquired cardiac magnetic resonance images may be used for radiomics analysis. There is no need for dedicated acquisition protocols. *Volume segmentation*: The areas to be analysed are contoured. This may be a single area (e.g. region of suspected abnormality in the myocardium) or multiple areas. In this example the left (blue) and right (red) ventricular blood pool regions, and the left ventricular myocardium (green) have been segmented and will be analysed. *Radiomics feature extraction*: Radiomics features are extracted from the segmented region of interest. *Feature selection*: Features that are most robust and informative are selected from the extracted features using methods such as clustering and principal component analysis. *Model building*: The selected radiomics features are used as predictor variables to build statistical models for disease discrimination or outcome prediction. Models are built using a training set with labelled training examples. *Diagnosis, risk stratification*: Models undergo internal and external validation and may ultimately be incorporated into clinical care for improved diagnostic accuracy and/or outcome prediction.

Once we have identified the final set of radiomics features through feature selection, we can begin to build our classification model (*Figure 7*). The predictor/discriminatory variables will be the radiomics features (input) and the output will be the desired label (e.g. HCM vs. healthy subject). To build the model, we require a sample (training set) of example cases (training examples) with known inputs and outputs, from which we have extracted and selected our features (*Table 1*). In some cases, logistic regression will be adequate to address a simple classification problem. More commonly, machine learning algorithms are used to train different models, from these, the model with the best performance is selected. Support vector machines (SVMs) are a commonly used machine learning algorithm for addressing classification problems with capability of modelling both linear and non-linear (SVM with kernels) relationships. The SVM identifies all the hyperplanes that could separate the different classes within the training set (e.g. HCM vs. healthy) and selects the one that maximizes the margins between classes (*Figure 6*). Other commonly used algorithms include decision tress and random forests.

**Table 1:** Example of training set for model building^a^

Training example	Output (label)	Input (radiomics features)
1	Hypertrophic cardiomyopathy	*x_1_, x_2_, x_3_, x_4_, … x_n_*
2	Hypertrophic cardiomyopathy	*x_1_, x_2_, x_3_, x_4_, … x_n_*
3	Healthy	*x_1_, x_2_, x_3_, x_4_, … x_n_*
…	…	…
*n*	Healthy/hypertrophic cardiomyopathy	*x_1_, x_2_, x_3_, x_4_, … x_n_*

a In order to build a radiomics predictor model a training set is required. The trainings set is a sample of example cases (or training examples), which are correctly labelled with the desired model output (e.g. hypertrophic cardiomyopathy) and have CMR images available. Radiomics features are extracted from the CMR images of all example cases in the training set. From these, a reduced number of features is selected, limiting to features that are most robust and informative, which are taken forward for model building often using machine learning algorithms. The algorithms determine how much weight (importance) is placed on each feature to achieve optimal model performance. The model developed from the training set should then undergo internal validation with a sample of cases which has not mixed with the training set.

### Validation

The classification accuracy of the model built using the training set should be assessed on an internal dataset that has not mixed with the training data during the model building or feature selection process. External validation with an independent external dataset is important for assessment of model performance and generalizability. The models are able to output a probability of belonging to a class and not only a discrete value. Model performance is thus assessed using measures of sensitivity, specificity, receiver operating curves, and area under the curve (AUC). Noting high inconsistency in reporting of multivariate classification tasks in medicine, researchers should take care to follow guidelines set forth by TRIPOD (Transparent Reporting of a multivariable prediction model for Individual Prognosis Or Diagnosis) and the Radiomics Quality Score.^36^^,^^37^

### Clinical implementation

The main overarching motivator driving radiomics analysis is that certain radiomics features will correspond to particular disease states, and therefore, once identified, blueprints of radiomics features (radiomics signatures) may be used to accurately classify disease entities and clinical outcomes.

## Literature review

Several studies have demonstrated the feasibility and potential clinical utility of CMR radiomics and CMR texture analysis. In a small, proof-of-concept study Baessler *et al*.^12^ demonstrate the ability of CMR texture analysis to accurately differentiate between myocardial disease states and healthy hearts from still non-contrast cine images. They report significant differences in texture parameters of individuals with HCM and healthy controls (*n *=* *32, *n *=* *30, respectively). They identify GLevNonU (Grey-level non-uniformity), a parameter derived from the GLRLM indicative of high heterogeneity, as the best discriminator of the two subgroups. Their findings suggest, that in addition to accurate disease classification, radiomics analysis may have value in reflecting alterations of the myocardium at a tissue level. In support of these suppositions, Cetin *et al*.^13^ demonstrate the ability of radiomics analysis to detect alterations in myocardial architecture that are not apparent through visual inspection of CMR images. The presented radiomics model was able to discriminate with good accuracy (AUC 0.76 ± 0.13) between the hearts of individuals with hypertension, but apparently normal hearts, and those of healthy controls. The 11 radiomics features selected for inclusion in their model were LV texture features (vs. shape features), supporting the idea that individuals with hypertension have subtle changes at the myocardial level, which may be detected with radiomics analysis but not through existing image analysis techniques.

In addition to differentiating disease from healthy states, studies have demonstrated the ability of radiomics models to make more challenging distinctions between different disease states. For instance, Neisius *et al*.^14^ demonstrate that radiomics analysis applied to native T1 maps provides incremental classification accuracy over global T1 measures in distinction of HCM from hypertensive heart disease. In another study, Baessler *et al*.^15^ demonstrate the superior diagnostic accuracy of radiomics texture analysis applied to T1 and T2 maps in discriminating biopsy proven infarct-like acute myocarditis in comparison to mean T1, mean T2, and Lake Louise diagnostic criteria.

Assessment for myocardial infarction and myocardial viability are two major strengths of CMR and account for a substantial proportion of clinical CMR referrals. Several radiomics studies have demonstrated the possibility of making such clinical distinctions through analysis of gadolinium-free images. This is a highly attractive prospect both from a safety and time efficiency perspective. Baessler *et al*.^16^ demonstrate the possibility of accurately distinguishing individuals with myocardial infarction from healthy controls through texture analysis of non-contrast cine images. Similarly, Larroza *et al*.^17^ were able to discriminate non-viable myocardium (as per late gadolinium enhancement, LGE) using texture analysis of non-contrast cine images. In another study, Larroza *et al*.^18^ demonstrate the ability of texture analysis to accurately identify myocardial infarction from non-contrast cine images, in cases where the infarction was mostly visually imperceptible. Further to this, they demonstrate the ability to distinguish acute myocardial infarction (occurring within 1 week) from chronic myocardial infarction (occurring >6 months prior to imaging) from radiomics analysis of LGE CMR images.

Limited studies report on the ability of radiomics analysis in predicting important clinical outcomes. In a study of 34 individuals with chronic myocardial infarction, Kotu *et al*.^19^ demonstrate that textural features extracted from LGE scar provide incremental value over scar size and location in determining the risk of life-threatening arrhythmias. Amano *et al*.^20^ demonstrate differences in textural features of LGE images in HCM patients with a history of ventricular tachycardia compared to those without history of arrhythmia. Furthermore, Cheng *et al*.^21^ demonstrate strong association of LGE textural features with a composite of several adverse clinical outcomes (including death and life-threatening arrhythmias) in individuals with HCM and reduced LV systolic function.

These studies demonstrate the potential of CMR radiomics to augment current image analysis approaches and provide superior disease classification and prognostication. The possibility of deriving from radiomics analysis of non-contrast images equivalent information to gadolinium-enhanced images is a hugely desirable prospect. Furthermore, the limited CMR radiomics literature and the more extensive work from oncology suggest that decoding image phenotype through radiomics texture analysis may provide additional value in reflecting pathology at the tissue level, with the potential to provide insights into disease pathophysiology.^38^ However, there are many limitations to these studies which must be addressed in future work to allow drawing of robust conclusions.

## Challenges and directions for future research

There are many sources of heterogeneity in acquisition and post-processing of CMR images occurring at all stages of the workflow such as, scanner vendor, position and number of receiver coils, planning and positioning of cut-planes, pulse sequence variables [flip angle, relaxation time (TR), echo time (TE), field of view, slice thickness], and many more. Whilst these variations do not alter images to a degree that would impact current image analysis techniques, they become highly significant when we attempt to numerically quantify all aspects of the image as in radiomics. It then becomes difficult to ascertain whether differences in radiomics features reflect biological differences or variations in image acquisition and processing. Lack of reproducibility presents a serious problem for radiomics and limits the reliability of radiomics models. A potential solution would be to promote standardization of image acquisition and post-processing. This would be extremely challenging due to the high degree of variability at all levels of image acquisition, reconstruction, and post-processing. Imposing strict uniformity to this scale is impractical. Therefore, for radiomics to transition to routine practice, it must adapt to existing practices. Uncertainty about reproducibility of radiomics features and hence the reliability of models constructed from these features is one of the most important challenges for radiomics. There is urgent need to identify radiomics features that are robust to real-life variations in CMR images, so that future studies may prioritize inclusion of these features in radiomics models. Furthermore, there remain unanswered technical questions surrounding size dependency and need for normalization of radiomics features, particularly in the setting of whole organ radiomics as with CMR.

An important limitation of existing CMR radiomics studies is absence of external (and sometimes also internal) validation of the proposed models. Therefore, we cannot comment on the generalizability of these findings. It is likely that the performance of these models applied to external datasets will be extremely poor, due to both heterogeneity in image acquisitions and the small size of the training datasets. The small samples used in these studies do not adequately capture the variation in phenotype of studied conditions. For example, there are many phenotypic variants of HCM that would not be covered in existing sample sizes. Therefore, there is need to build models on large high-quality training datasets and to demonstrate generalizability through meticulous internal and external validation.

Performance of existing clinical models for prediction of important outcomes such as death, or life-threatening arrhythmias is largely inadequate. Whilst there is value in the radiomic models as diagnostic tools, there is greater need for better prediction of important clinical outcomes. The potential superior risk stratification of radiomics models is reported with paucity in the literature and represents an important knowledge gap. In addition to disease/outcome classification models, radiomics texture features may reflect characteristics of the myocardial architecture and as such provide insights into disease pathophysiology. Therefore, there is value in linking what is seen on radiomics analysis with tissue histopathology and studies reporting such findings would be of great interest. As radiomics may be applied retrospectively to previously acquired images, such studies may be carried out on existing datasets that have paired imaging-histopathology data.

Finally, radiomics models would be greatly enhanced by incorporation of relevant clinical data, biomarkers, and genomics. Integration of data in this way can facilitate development of powerful tools for personalized medicine, which serves as an ambitious, but achievable aim. To enable development of such approaches we must ensure appropriate infrastructure within research and clinical teams to support the high computational power required to build and implement these all-encompassing clinical models. Furthermore, storage and access to these large volumes of data must be safeguarded vigilantly through purpose-built security systems.

## Conclusion

Radiomics presents a novel quantitative image analysis technique with potential to greatly augment CMR phenotyping in a manner that enhances our diagnostic and predictive capabilities. CMR radiomics features may also provide unique insights into pathophysiology at the tissue level aiding understanding of disease mechanisms. However, existing studies are limited to small select datasets and there are many unresolved technical challenges. The availability of big data and high computational power mean that addressing these challenges is achievable through an organized approach with positive interdisciplinary collaborations.

## Funding

This article is supported by the London Medical Imaging and Artificial Intelligence Centre for Value Based Healthcare (AI4VBH), which is funded from the Data to Early Diagnosis and Precision Medicine strand of the government’s Industrial Strategy Challenge Fund, managed and delivered by Innovate UK on behalf of UK Research and Innovation (UKRI). Views expressed are those of the authors and not necessarily those of the AI4VBH Consortium members, the NHS, Innovate UK, or UKRI. Z.R.E. was supported by a British Heart Foundation Clinical Research Training Fellowship (FS/17/81/33318). A.J. was supported by a Fulbright Predoctoral Research Award (2019-2020). S.E.P. acknowledges support from the National Institute for Health Research (NIHR) Cardiovascular Biomedical Research Centre at Barts NHS Trust and has received funding from the European Union’s Horizon 2020 research and innovation programme under grant agreement No 825903 (euCanSHare project). S.E.P. acknowledges support from the ‘SmartHeart’ EPSRC programme grant (www.nihr.ac.uk; EP/P001009/1). S.E.P. also acknowledges support from the CAP-AI programme, London’s first AI enabling programme focused on stimulating growth in the capital’s AI Sector. CAP-AI is led by Capital Enterprise in partnership with Barts Health NHS Trust and Digital Catapult and is funded by the European Regional Development Fund and Barts Charity. SEP also acts as a paid consultant to Circle Cardiovascular Imaging Inc., Calgary, Canada and Servier.

**Conflict of interest:** none declared.

## Supplementary Material

jeaa028_Supplementary_Data
