# Supplementary material for: Cardiac magnetic resonance radiomics: basic principles and clinical perspectives
Source: Eur Heart J Cardiovasc Imaging. 2020 Mar 6;21(4):349–56. doi: 10.1093/ehjci/jeaa028 (PMC7082724; doi:10.1093/ehjci/jeaa028)
Supplement: jeaa028_Supplementary_Data [file jeaa028_supplementary_data.zip › jeaa028-suppl_data/radiomics_Supplementary Table 1.docx]

**Supplementary Table 1. Selected radiomic features from texture analysis (second-order statistics)**

GLCM: Grey level co-occurrence matrix; GLRLM: Grey level run-length matrix; GLSZM: Grey level size zone matrix; GLDM: Grey level difference matrix; NGTDM: Neighbouring grey tone difference matrix.

| **GLCM** | **GLRLM** | **GLSZM** | **GLDM** | **NGTDM** |
| --- | --- | --- | --- | --- |
| Homogeneity | Short run emphasis | Small area emphasis | Small dependence emphasis | Coarseness |
| Dissimilarity | Long run emphasis | Large area emphasis | Large dependence emphasis | Contrast |
| Contrast | Grey-level non-uniformity | Grey-level non-uniformity | Grey-level non-uniformity | Busyness |
| Correlation | Run length non-uniformity | Size zone non-uniformity | Dependence non-uniformity | Complexity |
| Autocorrelation | Run percentage | Zone percentage | Grey-level variance | Strength |
| Joint average | Grey-level variance | Grey-level variance | Dependence variance |  |
| Cluster prominence | Run variance | Zone variance | Dependence entropy |  |
| Cluster shade | Run entropy | Zone entropy | Low grey-level emphasis |  |
| Cluster tendency | Low grey-level run emphasis | Low grey level zone emphasis | High grey-level emphasis |  |
| Difference average | High grey-level run emphasis | High grey level zone emphasis | Small dependence low grey-level emphasis |  |
| Difference entropy | Short run low grey-level emphasis | Small area low grey-level emphasis | Small dependence high grey-level emphasis |  |
| Difference variance | Short run high grey-level emphasis | Small area high grey-level emphasis | Large dependence low grey-level emphasis |  |
| Joint energy | Long run low grey-level emphasis | Large area low grey-level emphasis | Large dependence high grey-level emphasis |  |
| Joint entropy | Long run high grey-level emphasis | Large area high grey-level emphasis |  |  |
| Inverse difference moment |  |  |  |  |
| Inverse difference |  |  |  |  |
| Inverse variance |  |  |  |  |
| Maximum probability |  |  |  |  |
| Sum average |  |  |  |  |
| Sum entropy |  |  |  |  |
| Sum of squares |  |  |  |  |
