# Supplementary material for: Cardiac magnetic resonance radiomics: basic principles and clinical perspectives
Source: Eur Heart J Cardiovasc Imaging. 2020 Mar 6;21(4):349–56. doi: 10.1093/ehjci/jeaa028 (PMC7082724; doi:10.1093/ehjci/jeaa028)
Supplement: jeaa028_Supplementary_Data [file jeaa028_supplementary_data.zip › jeaa028-suppl_data/Supplementary Table 2_rad.docx]

**Supplementary Table 2. Summary of selected studies demonstrating value of CMR radiomics models**

| **Study** | **Disease studied and comparator** | **Images analysed** | **Model performance** | **Conclusion** |
| --- | --- | --- | --- | --- |
| Cetin et al. ^13^ | Hypertension with apparently normal hearts vs healthy controls | Still cine images in end-systole/diastole | CMR radiomics models had good discriminatory performance: (AUC 0.76 ± 0.13) | Radiomics analysis can distinguish hypertensive patients from healthy controls, even in the absence of evident HHD with conventional CMR indices. |
| Neisius et al. ^14^ | HCM vs HHD | Native T1 maps | ﻿ CMR radiomics texture models separated the two disease entities with high accuracy at 80.0% (c-statistic: 0.89; 95% CI: 0.77 to 1.00). Global native T1, had modest accuracy of 64% (c-statistic: 0.549; 95% CI: 0.452 to 0.640). | Radiomics texture models applied to native T1 maps discriminate between HCM and HHD with greater accuracy than global native T1. |
| Baessler et al. ^15^ | Biopsy-proven infarct-like acute myocarditis vs Biopsy-negative infarct-like acute myocarditis | T1 and T2 maps | **﻿**Mean T1 and T2 values and LLC showed a low diagnostic performance, AUC: 0.65 (95% CI: 0.45, 0.85) , 0.67 (95% CI: 0.49, 0.85) , and 0.62 (95% CI: 0.42, 0.79) respectively. ﻿Radiomics texture features model resulted in higher diagnostic performance, AUC: 0.88 (95% CI: 0.73, 1.00) (P <0.001). | Radiomics models from analysis of T1 and T2 maps discriminate between biopsy-proven and biopsy-negative infarct-like acute myocarditis with greater accuracy than mean T1, mean T2, or the LLC. |
| Larroza et al. ^38^ | Non-viable myocardium vs Viable myocardium | Still contrast free cine images | Using LGE findings as ground truth for infarction: ﻿Radiomics analysis of non-contrast image, (AUC): 0.849, sensitivity of 92% to detect non-viable segments, 72% to detect viable segments, and 85% to detect remote segments | Radiomics analysis can distinguish viable from non-viable myocardium with reasonable accuracy from contrast-free images. |

AUC: area under curve; CI: confidence interval; HCM: hypertrophic cardiomyopathy; HHD: Hypertensive heart disease; LGE: late gadolinium enhancement; LLC: Lake Louise Criteria. ^*^Adverse event: ﻿The end- point was the composite of cardiovascular death (cardiac death due to progression of heart failure or SCD), aborted SCD, heart transplantation and unscheduled heart failure ﻿hospitalization. SCD was defined as witnessed sudden death with or without documented ventricular fibrillation or death within 1 h of new symptoms or nocturnal deaths with no antecedent history of worsening symptoms
